# Encapsulation of Gold Nanorods with Porphyrins for the Potential Treatment of Cancer and Bacterial Diseases: A Critical Review

**DOI:** 10.1155/2019/7147128

**Published:** 2019-04-30

**Authors:** Nthabeleng Hlapisi, Tshwafo E. Motaung, Linda Z. Linganiso, Oluwatobi S. Oluwafemi, Sandile P. Songca

**Affiliations:** ^1^Department of Chemistry, University of Zululand, X1001, KwaDlangezwa, KwaZulu-Natal, South Africa; ^2^Department of Applied Chemistry, University of Johannesburg, P.O. Box 17011, Doornfontein, Johannesburg 2028, South Africa; ^3^Centre for Nanomaterials Science Research, University of Johannesburg, Johannesburg, South Africa; ^4^Department of Chemistry, University of Kwazulu Natal, Kwazulu Natal, South Africa

## Abstract

Cancer and bacterial diseases have been the most incidental diseases to date. According to the World Health Report 2018, at least every family is affected by cancer around the world. In 2012, 14.1 million people were affected by cancer, and that figure is bound to increase to 21.6 million in 2030. Medicine therefore sorts out ways of treatment using conventional methods which have been proven to have many side effects. Researchers developed photothermal and photodynamic methods to treat both cancer and bacterial diseases. These methods pose fewer effects on the biological systems but still no perfect method has been synthesized. The review serves to explore porphyrin and gold nanorods to be used in the treatment of cancer and bacterial diseases: porphyrins as photosensitizers and gold nanorods as delivery agents. In addition, the review delves into ways of incorporating photothermal and photodynamic therapy aimed at producing a less toxic, more efficacious, and specific compound for the treatment.

## 1. Introduction

Drugs based on metals have since been used during ancient times; however, modern metal-based medicine dates to about 50 years ago through the use of cisplatin; since then, the antitumor activity of these metals has been studied [1–5]. Gold salts and arsenic compounds have been used for the treatment of arthritis and syphilis, respectively. Heavy metals, rhodium, indium, palladium, and noble metals have the ability to be used as therapeutic agents. The noble metal nanoparticles work better when ligands are attached to them as opposed to when they operate singularly as free ligands; in addition, antibiotic compounds can also be bound to the metals, thereby increasing their efficacy or to avoid the resistance by drugs [6, 7]. In their elemental and coordinated states, metals act as various agents: antibiotic, antimalarial, antibacterial, antiviral, antitubercular, antimycotic, antiarthritis, and anti-inflammatory. In their elemental states, nanoparticle formulations are used as carrier agents for drugs and to toxify bacteria but this toxicity of bacteria in humans is still a subject of research [8]. The specificity and selectivity of target drugs that only hit the affected cells and tissue have been a challenge in the therapeutic field, but the use of receptor nanoparticles has been implemented to overcome this challenge. Other challenges as highlighted by Akhtar et al. [9] are primarily on the basis of the design of a nanocarrier, the drug loading efficiency, how stable the ligands are on the nanoconjugates, maximum receptor-ligand interactions, and the time the targeted receptor would be expressed, the toxicity of the nanoparticle, and immunity to the blood system [10]. Gold nanorods (AuNRs) are used in photodynamic and photothermal therapy for clinical purposes on the basis of their optical and chemical properties. Porphyrin is one of the common photosensitizers used for the treatment of tumors and antibacterial infections. Nanoparticles have been introduced to the clinical field as to prevent or assist in the traditional methods of treating cancer.

Nobel metal nanoparticles have strong electric fields at the surface; hence, the absorption and scattering of electromagnetic radiation by those nanoparticles are enhanced, making them ideal choices in the photothermal cancer therapy. The best option is to use agents that are active at the near-infrared region for minimal light extinction by the intrinsic chromophores in the tumor tissues. AuNRs with suitable aspect ratios have the ability to absorb and scatter light in the NIR (650–900 nm), hence used in molecular imaging and photothermal cancer therapy. Solid tumors have leaky blood vessels with cell junction gaps from 100 nm to 780 nm, and this permeable vasculature allows for the extravasation of gold nanoparticles into the tumor matrix. The optimal aspect ratio as experimentally obtained by Singh et al. for *ex vivo* experiments was determined as 4.0 ± 0.06 as per TEM results. The aspect ratio resulted in the SPR peak of the AuNR to be around 808 nm which is in the NIR and allows for deep optical tissue penetration [11].

Some scientists however argue for the use of nanoparticles as tumor-targeted delivery systems in the clinical environment. An example is seen where Van de Meel et al. [12] state that the “unappreciation” of nanomedicine is due to clinical trials practised on mice as opposed to humans and that only 0.7% of the researched nanomedication even reaches clinical trials. He however concluded that researchers should focus not only on the understanding of the biology and chemistry behind nanocarriers but also on the idealistic “disease-driven” approach on their potential.

The use of gold nanoparticles has been of interest lately due to their impeccable properties as carrier agents of drugs; the properties include the fact that a wide range of organic molecules can adhere and be bonded to the nanoparticles, they have low levels of toxicity, their absorption is very tunable, and they have small sizes and high surface area to the volume ratio. These properties however ideally work when the surface of the gold particles is modified and tuned to suit the use [13]. Gold nanoparticles are biologically inert, have high dispersity, are noncytotoxic, are biocompatible, and are optically tunable [14]. The treatment of cancer has traditionally made use of the systematic delivery of an anticancer agent which unfortunately leads to the accumulation of the agent to the tumor cell; a new method has however been developed which ensures that the anticancer agent is delivered through the lymphatic network. Oladipo et al. used the polymeric AuNRs (PAuNRs) to deliver drugs from an accessory lymph node to a proper auxiliary lymph node containing a tumor in order to treat lymph node metastases [15]. Coating of nanoparticles with alkane thiols monolayer has become of great attraction as resulting nanoconjugates have unique optical and electronic properties that depend and can be controlled by the particle size and the nature of the protecting molecules [16].

Gold nanoparticles possess unique optical properties. AuNRs particularly have two distinctive absorption bands: one is caused by light absorbed on the short axis (transverse) and another due to light absorbed on the long axis (longitudinal). The ability of the AuNRs to be optically controlled and the fact that they are very sensitive to changes in the local environment makes them excellent materials for sensing, photothermal therapy, and imaging [17]. AuNRs applications are vast; they can be used in biomedical technologies, plasmon-enhanced spectroscopies, and optical and optoelectronic devices. AuNRs exhibit special optical properties in cancer diagnostics and treatment. Their advantages however still bring a few questions: their toxicity in the blood system, their biodistribution, and their fate *in vivo*. Still to be explored is a further optical control of gene delivery and use of nanorods for *in vivo* spectroscopic tumor detection combined with organic dye molecules [18].

**Figure 1:**
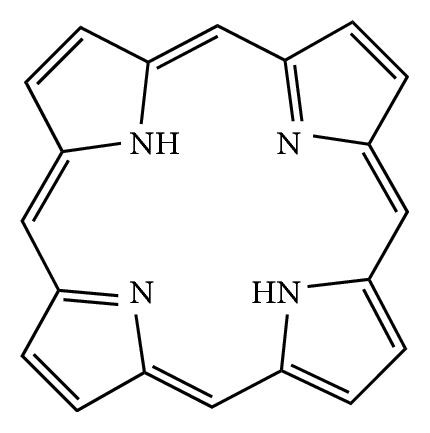
Structure of porphyrin.

Photodynamic therapy is one of the most effective regimens in the treatment of cancer, precancerous inductions, actinic keratosis, infections, dermatology, cardiovascular illness, and wound healing. For photodynamic therapy to successfully proceed, three elements have to be present: oxygen, a photosensitizer, and light [19]. Porphyrins are photosensitizers which selectively accumulate in tumor cells, releasing cytotoxic substances and hence destroy the cancerous cells with minimal harm to the surrounding healthy cells. Exposure of a photosensitizer to photoactivating light enhances the destruction of malignant tissues. Clinically, common photosensitizers used are the haematoporphyrin derivative (HPD) and the Photofrin II, which is a purified form of HPD [20]. Porphyrin is a naturally occurring heterocyclic compound shown in Figure 1 [21].

Heterocyclic compounds occur widely in nature in compounds such as hemes and chlorophylls. These compounds are used in medicines, pharmaceutical, agrochemical, and energy materials. Polyheteroatomic heterocycles are used in clinical therapy as they allow for direct interactions with the biological targets which are often not possible with single heterocyclic compounds [22]. Porphyrins are heterocyclic compounds with semiconducting properties; these compounds can be used for a number of applications: artificial photosynthesis, catalysis, molecular electronics, sensors, nonlinear optics, and solar cells. The functionality of porphyrins depends on their crystallography, which depends on the plane of the macrocycle and the conjunction of the pi-bonding to the adjacent macrocycle or how the macrocycles are interconnected with the ligands by covalent bonds. The modification of the basic porphyrin structure enhances its semiconductivity property and hence its ability to be used as a photosensitizer. Porphyrins also have the tumor-localizing ability [23].

Encapsulation of gold nanorods with porphyrin has been achieved before to obtain novel multifunctional nanoparticles. The porphyrin structures were, for example, doped with silica shells to protect them from the external bioenvironment [24]. The resulting multifunctional nanoparticles are good candidates for both photosensitization and two-photon imaging and also image-guided therapy.

The use of imaging therapy has many advantages over conventional therapy. The merits of two imaging therapy include the bioimaging that provides for deeper penetration (light) and has a 3D imaging capability. Metal nanoparticles especially gold nanorods have shown an excitingly good promise in two-photon imaging. They have high two-photon luminescence which is approximately 58 times greater than that of a single rhodamine molecule [25]. The biological transparency window is around 6000–10000 nm which coincides with the longitudinal plasmon band of the gold nanorods. The nanorods alone however lack the targeting ability, hence with combination with porphyrins will create a more specific selective multifunctional compound.

The purpose of this study is to revise the coating performed on nanorods with porphyrin for further modification for the treatment of cancer and bacteria using photodynamic and photothermal therapy [26–29].

## 2. Literature Review

### 2.1. Gold Nanoparticles

Generally, metal nanoparticles have been used for cancer diagnosis and treatment for the past decades [30–34]. Multifunctional nanoparticles have specifically been used for a number of aspects such as targeting, imaging, and therapy as a way to overcome challenges imposed by conventional treatment (radiotherapy, chemotherapy, and surgery). Amongst all, the use of iron oxide nanoparticles received overwhelming attention primarily due to super magnetic properties for magnetic resonance imaging (MRI). In fact, magnetic nanoparticles other than gold and carbon nanoparticles could be degraded to the respective metal cations in the body especially in acidic media, increasing the toxicity of the long-term nanoparticles residing in the body [35]. Certain metal nanoparticles interact with light, and that proved to be an opportunity for biophonic nanomedicine [36, 37]. One of the main fields that use nanoparticles (NPs) is the image-guided therapy; it uses multifunctional nanoparticles based on surface plasmon resonance absorption property which is tunable in the near-infrared region [38, 39]. Typical examples are gold nanorods, for which optical properties could be explained better by the Mie–Gans theory [40]. The sensitivity of the plasmon resonance frequency of the nanorods towards the refractive index of its surroundings makes them suitable for use in biological sensing. They are also proven to be very sensitive to thermal environments and possess nonlinear optical response properties [41, 42]. Synthesis of the nanorods is one of the main parameters that are controlled for optimization of properties [43]. An important technology investigated in this review as a promising factor for the image-guided therapy and diagnosis is the passivation of gold nanorods [44, 45].

Gold nanoparticles have different shapes ranging from spherical, suboctahedral, octahedral, decahedral, icosahedral multiple twinned, irregular shaped, tetrahedral, nanotriangles, nanoprisms, hexagonal platelets, and nanorods [46–48]. Fluorescent nanoparticles have good biocompatibility for molecular imaging and metabolites used for cellular functions in cancer. On the other hand, nanorods show special optical and chemical properties for biological applications. They also have unique anisotropic geometry for tuneable absorption in the visible and near-infrared region. These phenomena make them useful for biosensing, photothermal therapy, and gene delivery [49–52]. Generally, nanoparticles are used for their light scattering and absorbing abilities, and scattering is more important for microscopy and optical coherence tomography (OCT) [53].

The effects of the nanoparticles can be detected using various imaging apparatus: magnetic resonance imaging, nuclear imaging, and photoacoustic imaging. After the administration of the nanoparticle to the tumor cell, they land on a solid target tissue, and their activity can be changed by an external stimulus. An example is photothermal therapy, in which the light energy is converted to heat energy for damaging the cancer cells [54, 55]. The dependence of the excitation of the noble metal nanorod energy lies in the dimensions of the nanorods and its environment. This enhances tunable optical properties which can be demonstrated by the Mie–Gans theory. It states that there is a direct dependence of the geometry end cap of the nanoparticles and the particles' size, which affects the peak position in the absorption spectra. For AuNRs, this gives rise to the two absorption bands by the longitudinal band and the transverse band [56–58].

#### 2.1.1. Properties of Gold Nanorods

**Figure 2:**
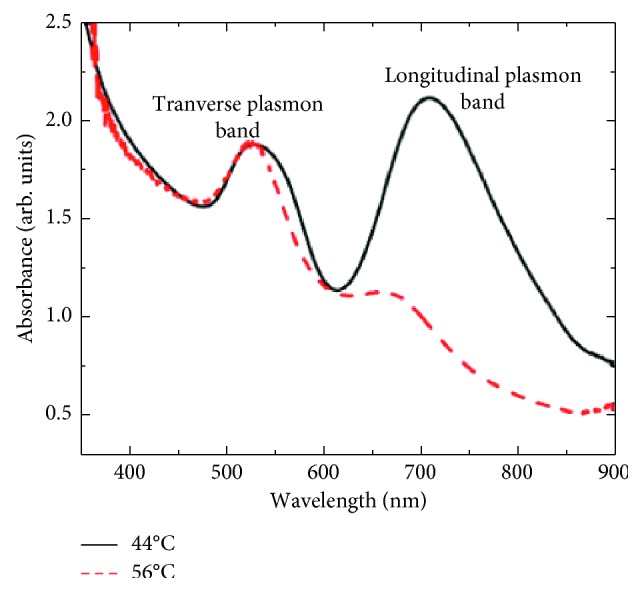
UV-Vis spectrum of AuNRs at different preparation temperatures [61].

**Figure 3:**
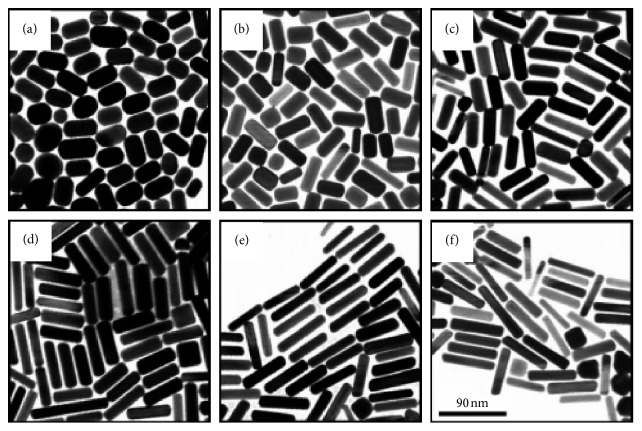
Illustration of gold nanorods of different aspect ratios in AgNO_3_ growth solution with concentration (a) 0.03, (b) 0.05, (c) 0.075, (d) 0.1, (e) 0.125, and (f) 0.15 mmol^−1^ [67].

The optical properties of AuNRs are of great importance and make them excellent to use in therapy. In the past, the use of gold nanoparticles was limited to additives for aesthetic purposes, but emerging studies indicated that the peculiar optical properties at nanoscale of the particles brought other dimensions. For instance, the increase in rod length of the gold nanorod increases the longitudinal band red shift and the extinction coefficient [59, 60], as shown in Figure 2 [60, 62–66]. As a result, the nanostructures are used in sensing, imaging, and photothermal therapy. One property which qualifies the nanorods to be used in as biological sensors is that their absorption band changes with the refractive index of the local material. This phenomenon allows for very accurate sensing. They are often identified by their aspect ratio and are cylindrical rods with a width less than 10 nm to over 40 nm and length to several nanometers; an example is seen in Figure 3. For one to fully comprehend the unique features of nanorods, the robust extinction coefficient for predicting the concentration at an absorbance has to be known. The AuNRs are effective for the detection of sequences of infectious agents for diseases like HIV-1 [68].

AuNRs absorb at the near-infrared region where the maximum radiation through the tissue occurs, hence used for *in vivo* imaging and photothermal therapy. Nanorods exhibit two bands of surface plasmon resonance (SPR), which are a product of the conduction band along their long and short axes. On the visible region, the transverse band and the longitudinal band occur on the near-infrared region (NIR) [69–72]. Tailoring of the nanorods during synthesis enables the absorption bands to be of desired wavelength in the near-infrared region, and this can be attributed to the fact that the longitudinal length of the nanorods can be tuned via its aspect ratio. Their luminous energy can be converted into heat energy which is influenced by the maximum penetration of light in tissues favorable for photothermal therapy [73–76]. AuNRs have a higher distinct local field enhancement resulting in a significant surface-enhanced Raman spectroscopy (SERS) activity. Their modifiable surface area enables the incorporation of drugs for use as drug delivery agents [77–79].

The distinctive optical and electrical properties of AuNRs depend on size and aspect ratio. For the nanorods to efficiently work, several aspects need to be addressed: improvement of the synthesis of AuNRs in terms of reproducibility and efficiency, full understanding of the direct characterization of nanorod geometry functionalization, and lastly, to find a cost-effective and sensitive method that would be used for nanorod sensing. Another important aspect is coating which also depends on synthesis that may lead to covalent or noncovalent bonds at the interface [80–82].

**Figure 4:**
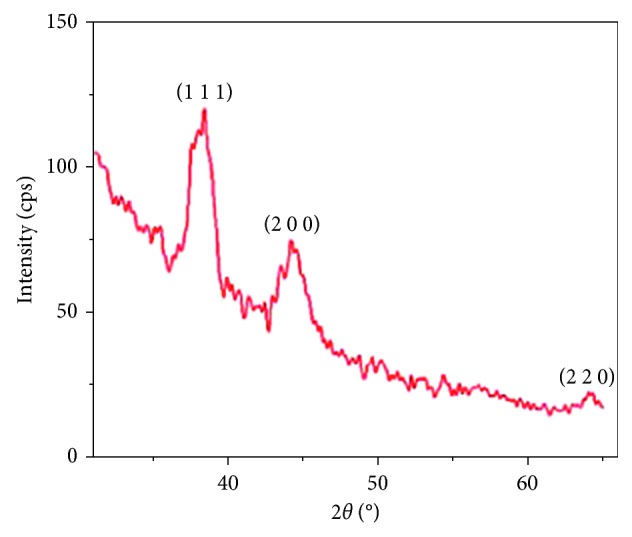
XRD pattern of gold nanorods as prepared by the sonoelectrochemical method [83].

X-ray diffraction (XRD) shows a face-centred cubic close packing arrangement of gold nanorods in specific areas. The XRD peaks are very consistent with ones of metallic gold. Sharp peaks resembling ones of gold are observed in Figure 4, hence confirming the crystalline nature of the gold nanorods. Figure 4 shows an XRD pattern of gold nanorods [83].

Various diffraction patterns are presented at 2*ð* with values 38.4°, 44.4°, and 64.6° which correspond to the following diffraction planes: (9111), (200), and (220), respectively. The peaks perfectly index to a cubic face-centred structure of a gold metal. The XRD showed a lattice constant of *α* = 4.082 Ă which is in agreement with 4.079 Ă, which is the standard diffraction pattern of a cubic gold metal (CAS: 7440-57-5) by Pallares [83].

#### 2.1.2. Synthesis of Gold Nanorods

**Figure 5:**
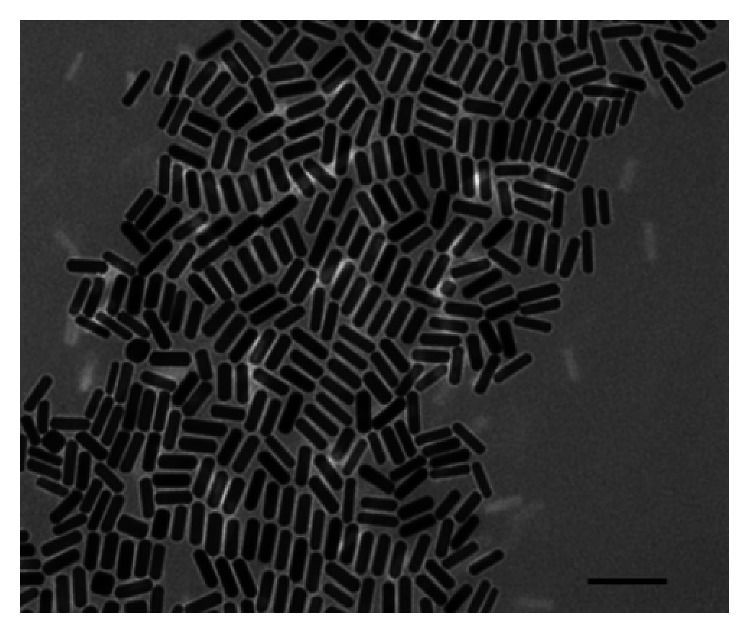
AuNRs prepared in the presence of a direction agent CTAB. Scale: 100 nm (TEM micrograph).

**Figure 6:**
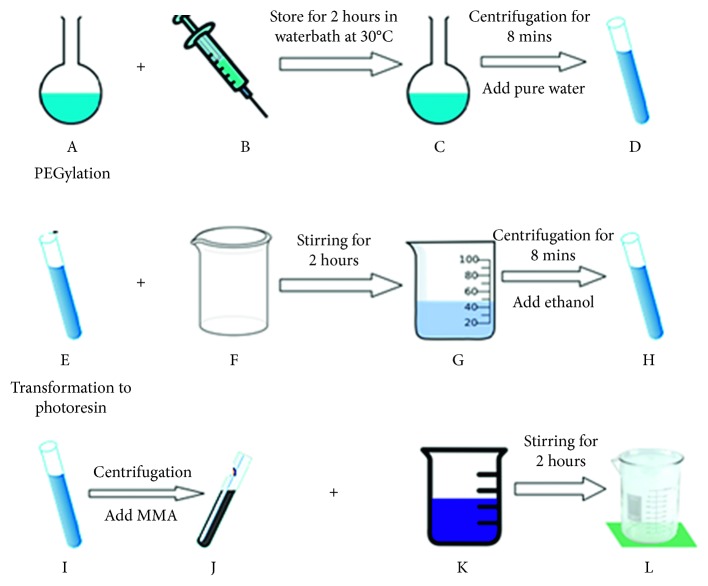
(A) Growth solution: CTAB, HAuCl_4_, AgNO_3_, H2SO_4_, and AA. (B) Seed solution: CTAB, HAuCl_4_, and NaBH_4_. (C) Gold nanorods dispersed in pure water. (D) Redispensation of gold nanorods in pure water. (E) Gold nanorods dispersing in water. (F) mPEG-SH solution. (G) Gold nanorods wrapped in mPEG-SH, in pure water. (H) Gold nanorods wrapped in mPEG‐SH, dispersing in ethanol. (I) Gold nanorods dispersing in ethanol. (J) Gold nanorods dispersing in MMA. (K) Photopolymerisation resin. (L) Gold nanorods dispersing in photoresin.

The nanorods are mainly synthesized using the wet chemical method and the hard template directed (an example in Figure 5) method [84, 85]. The wet chemical techniques involve the reduction of metal ions at gold surfaces in the presence of various surfactants; the technique however produces hybrids of nanospheres and other shapes clearly dominated by rods and that affects the ideal optical response of the gold nanorods. For high purity nanorods, the hard template directed methods are utilized by making use of the polycarbonate membrane and the anionic aluminum oxide (AAO) as templates [86, 87]. A typical example of the wet chemical method is the synthesis of AuNRs by seeding. In this method, a spherical seed of nanoparticles is added to a gold salt solution containing ascorbic acid, silver nitrate, and cetyltrimethylammonium bromide (CTAB) which then produces nanoparticles with a rod-like shape, as shown in Figures 5 and 6 [88]. CTAB enhances the rod-like shape of nanoparticles by preferably binding to the sides of the nanoparticles, and the concentration of gold nanoparticles is reduced slowly by the growth of the nanorods particles. Variation of silver nitrate concentration is used for the alteration of the rod length. The synthesis is followed by the centrifugation with distilled water to purify the nanoparticles by removing excess CTAB which is cytotoxic, unreacted metal ions, and the ascorbic acid.

The hard template directed method includes photochemical and electrochemistry methods, from which nanorods are grown in an electrolyte solution under regulated current between two electrolytes [89–92]. A gold metal plate acts as an anode and a platinum plate as a cathode. Both electrodes are immersed into an electrolytic solution containing a cationic surfactant.

**Figure 7:**
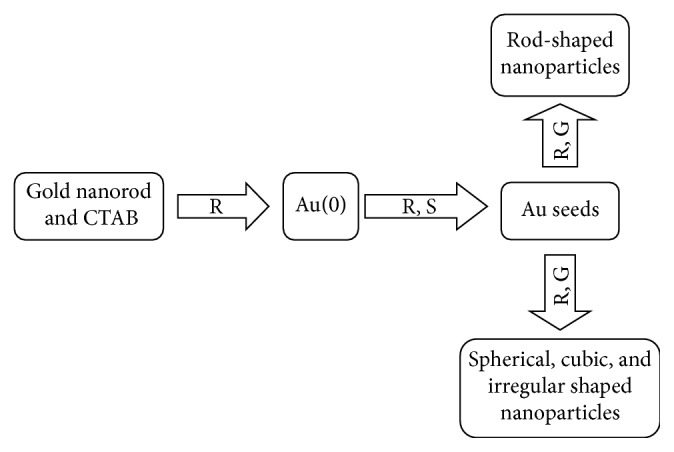
Illustration of the template method to synthesize gold nanorods and reduction of Au(I). S, forming Au seeds; G, growth of the Au seeds [93].

The solution normally is placed in an ultrasonic bath to avoid aggregation of the gold nanoparticles before the addition of acetone and cyclohexane to the electrolyte solution. Another electrochemical method can be used where the metal (gold) can be electrochemically deposited inside a nanoporous polycarbonate template of alumina membranes, as shown in Figure 7 [94]. In photochemical methods, gold salt is irradiated with UV light in the presence of CTAB and tetradodecylammonium bromide.

#### 2.1.3. The Seed-Mediated Method/Green Method Synthesis

**Figure 8:**
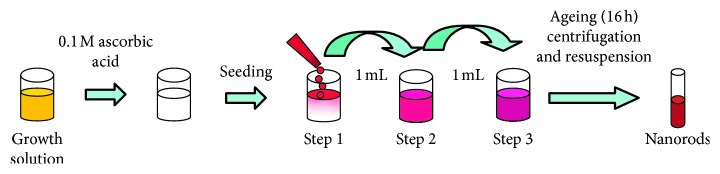
The general scheme used for the three step seed-mediated method [95].

Although the synthesis of AuNRs using the template method has been perfected and produces the AuNRs, other methods are also used as greener methods to give comparable results as the template method. Gole and Murphy [95] used the seed-mediated method as the alternative route to the template method. In this method as summarized in Figure 8, gold seeds of size 3-4 cm are initially synthesized by chemical reduction using a strong reduction agent (sodium borohydrite) in the presence of a capping agent (citrate). Following the step, the seeds are added to the solution of more metal salt, a weak reducing agent, and a surfactant directing agent (CTAB). The method generally results in monodisperse, stable gold nanorods with different aspect ratios.

Moreover, another alternative green method is to synthesize gold nanorods without the use of CTAB but using gelatin as a capping agent. The method was reported to have resulted in higher media stability and enhance photostability [96].

#### 2.1.4. Characterization of Gold Nanorods

Recent developments are done to develop nanotechnologies for specific drug delivery and multimodal activities [96–98]. Due to their distinctive chemical, physical, and photonic properties, AuNRs have been used for cancer therapy, diagnostic, and therapeutic applications. Ali et al. stated that the low resolution from some equipment limits crucial information about the nanorods [99]. Many researchers characterised the nanoparticles by using electron microscopy (EM), atomic force microscopy (AFM), dynamic light scattering (DLS) and static light scattering (SLS), X-ray diffraction (XRD), and the Fourier-transform infrared spectroscopy (FTIR), as indicated in Table 1 [112–114].

Table 1 shows the properties, synthesis, characterization, and applications of AuNRs.

#### 2.1.5. Application of the Gold Nanorods

A lot of studies indicated that the nanoparticles have the potential to be consumed as drug carriers, used in the detection and treatment of tumors, monitoring of treatment response, and to guide the therapeutic regimens [115, 116]. In addition, plasmon resonant gold nanorods are used as multifunctional agents for image-guided therapies. As the carriers, they promote the circulation time of drugs to take longer and improve and ensure that drugs do not degrade until they reach their targets. In the event of a tumor, the drug would land on the target still effective for an uptake through permeability, retention effect, and the receptor-mediated endocytosis. The large surface to volume ratio of nanocarriers helps them carry agents for chemotherapy, antiangiogenic, or gene therapy delivered to tumor sites for enhanced treatment [117, 118].

The versatility of nanoparticles enables them to be used in various ways. For example, in therapy, one way is for the treatment of ovarian cancer. This required a coat with thio-glucose, sensitizers to produce ROS to damage the cancer cells [119–122]. The use of the nanorods over nanospheres or any other nanoshapes is also due to narrower line widths at more or less the same resonance frequencies because of reduced radiation-damping effects. In fact, nanorods have the ability to emit two-photon luminescence (TPL) signals that are used for single photon detection, which is suitable for biological imaging purposes [123–125].

The use of AuNRs however has a few challenges. For example, under intense illumination, the shape of the rod changes to a nanosphere and longitudinal NIR resonance loss. This normally happens in the photothermal therapy. A coating with thermally stable material was effective to overcome the shape change [100, 101, 126]. The modifications of gold nanoparticles for clinical value are of great importance, taking note of uptake and cell targeting. Theranostic systems have been founded from many building blocks, including hybrid, organic and or inorganic nanoparticles, superparamagnetic iron oxide, and plasmonic gold nanoparticles, which have been extensively studied due to their unique physical properties. The hybrid of these particles can be used for *in vitro* or *in vivo* imaging, magnetic targeting, and photothermal therapy. The coating/passivation or functionalization of the gold nanoparticles has currently received enormous attention due to more multifunctional competitive applications [102, 103].

#### 2.1.6. Passivation of Gold Nanorods

**Figure 9:**
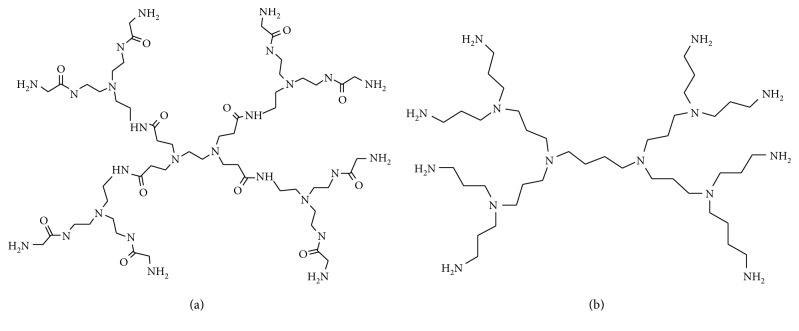
(a) A G1 PAMAM dendrimer and (b) G1 PPI dendrimer.

The significance of magnetic nanoparticles has been growing due to use in more important and exciting applications like use in biomedical diagnosis, catalysis, and photoluminescent materials. Due to their unstable nature to the surrounding environment, their applications are inhibited as they readily oxidise on exposure to air due to their large surface areas. Methods have been developed to increase oxygen resistance on metal nanoparticles such as plasma spraying, phosphating, and electrolyte deposition [104–107]. Consideration has to be made when encapsulating metal nanoparticles so as not to increase the thickness of the metal and hence interfering with the bulk metallic properties of the metal. One of the most ideal methods of passivation is the atomic layer deposition (ADL) method, which provides an ultrathin layer on the metal nanoparticle [108–111]. This method however does not guarantee the conformation of the geometry of the nanoparticles, hence only excellent for application in which precise morphology is not required [127, 128]. One of the methods used for the stabilization of metal nanoparticles is the attachment to dendrimers, ensuring and controlling the stability, size, and solubility of nanoparticles in a range of less than 1–5 mm diameter [129–131]. Examples of dendrimers include PAMAM and PPI as shown in Figure 9 [132]. Ligands and polymers have been used for the past years for the stabilization of metal nanoparticles and their application in catalysis and biocatalysis; in particular, their use with gold nanoparticles has been reported to yield excellent results.

The optical properties of AuNRs can be changed by slight changes in the shape or size of the nanorod; hence, it is important to ensure a stable environment for them. Sensing, imaging, and biomedical applications of AuNRs all come as a consequence of the ability of the AuNRs to be tunable, which is attributed to their optical properties contributed by their rod-like shape. Murphy et al. elaborates on the interfacial chemistry of nanorods [133]; three interfaces occur on the surface of nanorods, the gold-surfactant interface, hydrophobic surfactant bilayer, and a surfactant interface. These interfaces help in altering the nanorod properties in terms of stability, against aggregation and toxicity and how easily they can be assembled. Lastly, the solvent accessible interface provides a platform or directs how the nanorod can interact with other particles, macromoles, and living cells [134–136].

**Figure 10:**
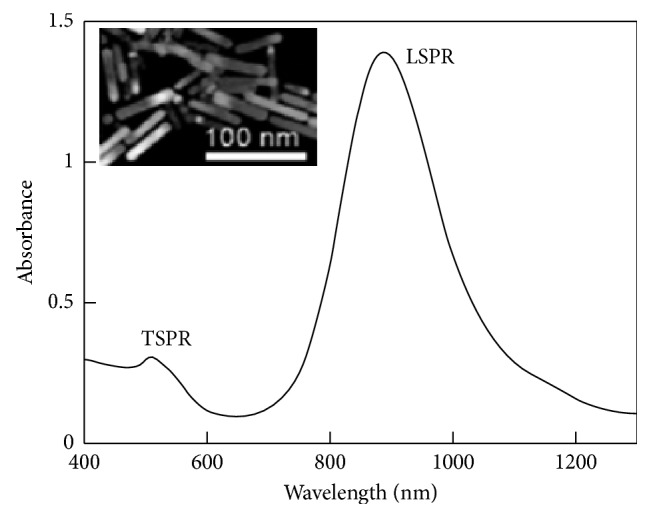
PC-AuNRs absorption spectrum, 0.5 nM Au nanorods (TEM image of PC-NRs).

A common method of the synthesis of AuNRs uses CTAB, which makes the functionalization of AuNRs using ligands a challenge. During the synthesis using this method, a double layer of CTAB is formed for the passivation; this layer is a problem in terms of bioconjugation, adsorption of DNA nonspecifically, cytotoxicity, and the stability of the nanorod and hence limits the use of these AuNRs for biological applications [137–140]. Absorption associated with AuNRs enables them to be used for dark-field light scattering observation or biological media. Even though CTAB is very toxic to the biological environment, complete removal from the AuNRs would yield unstable AuNRs in colloidal dispersion forming aggregates. To obtain functional AuNRs, CTAB has to be replaced by amphiphilic molecules. Cationic cerasone-forming lipids and cationic nonsililyated lipids are used to passivate AuNRs [141]. Moreover, the amphiphilic molecules were added as to overcome the shortcomings brought by CTAB especially the fact that it has high cytotoxicity preparation of phosphatidylcholine passivated AuNRs (PC-AuNRs) which have low cytotoxicity and efficiently used as photosensitizers using pulsed light [142]. The pulsed light however reshapes the nanorods into nanospheres, hence no absorption spectra at the near-IR region. This helps in the damage of only tumor cells not healthy cells even with successive irradiation. The combination of photosensitizers and light induces the selectivity for tumors from healthy cells and also use of light produces heat just around the photosensitizer, hence the destruction of cells.  Figure 10 [119] shows the absorption spectra of a PC-AuNRs in solution. It shows a normal NR spectrum with two SP peaks: ∼900 nm for the LSPR and ∼520 for the TSPR.

Ferric acid at room temperature as an etching agent for AuNRs is also used to passivate gold nanorods. The method used decreased the length of the AuNRs but not their diameter due to the oxidation of AuNRs by ferric ions, the shortening of only the length helps in the provision of the nanorod's desired aspect ratios and selective optical and also selectively removes other nanostructures. The shortening in length of the nanorods was monitored by TEM and the UV-vis absorption spectroscopy, and the results showed the reduction of electron potential of the gold species by halide ions and acceleration in oxidation of the AuNRs by ferric ions [121].

**Figure 11:**
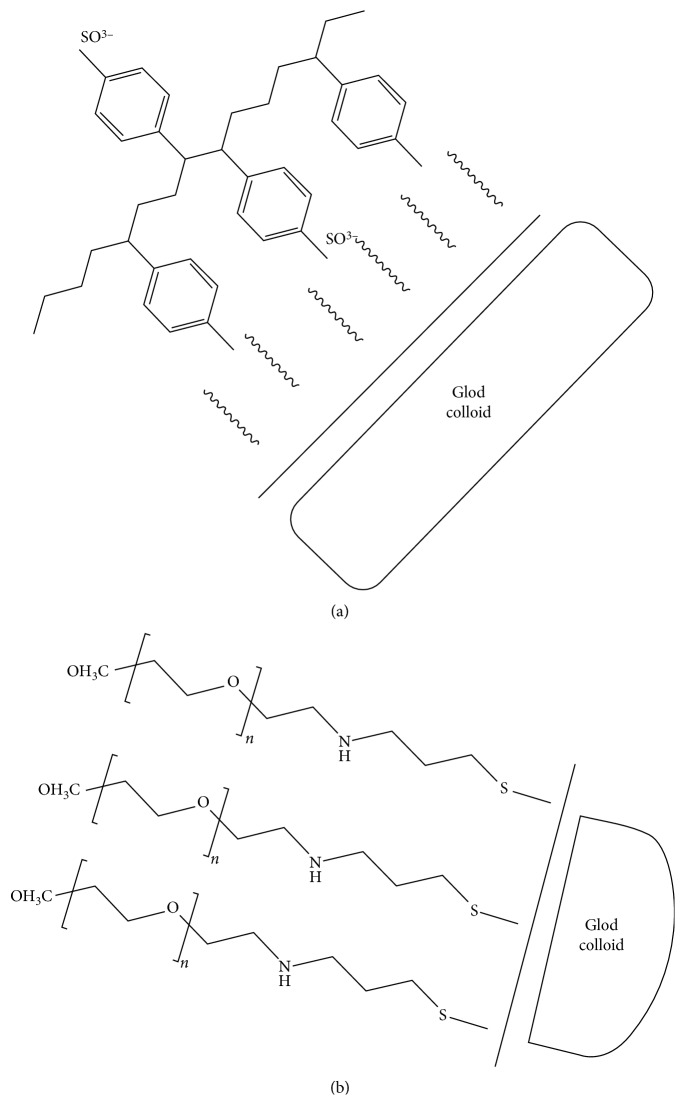
Surface modification of AuNRs. (a) PSS, encapsulation occurs on the CTAB bilayer through electrostatic adsorption. (b) mPEG-SH, replacement of the CTAB by PEG by the gold sulphur binding [123].

Other ways to encapsulate the particles include polyelectrolyte coatings such as poly(diallyldimethylammonium chloride) (PDADMAC), poly(4-styrenesulfonic acid) (PSS), poly(acrylic acid) (PAA), or poly(allyamic) hydrochloride (PAH); these molecules reduce the interaction of CTAB with cells. PEGyated AuNRs are also used, which to some extent replace the CTAB in the molecule hence reducing toxicity [122].  Figure 11 illustrates the encapsulation with polymer-based molecules.

The treatment of particles with PEG-SH replaces the CTAB in the molecules which is toxic, replaces it with PEG (polyethylene glycol), and it also enhances the particles to be more stable under different conditions. Takahashi et al. stated that the PEG as a linker to attach antibodies is still a field to be explored further [91]. Lastly, a hydrophobic polymeric precursor, polyvinyl acetate (PVA) which changes into polyvinyl alcohol which is hydrophilic, is used to replace the nonbiocompatible and toxic CTAB [143].

AuNRs perform adequately in their retained shape; hence, a suitable coat has to be used for such. CTAB is the main inhibitor in AuNRs application due to instability; hence, thiol monolayers are used which provide for better stability and organic media for compatibility [144]. Polymer (including porphyrin) coated nanorods are used as either ionic or cationic molecules with different charge densities, and these also give a promising future as biological delivery agents [145, 146].

#### 2.1.7. The Replacement of Quantum Dots over Gold Nanorods

Quantum dots (QDs) are nanocrystals with semiconducting properties. These materials comprise elements from Group II to VI or III to V, and their sizes range from 2 nm to 19 nm. QDs such as AuNRs have unique optical and chemical properties [147]. These materials have an excellent future in biomedical imaging and detection; however, due to the heavy metals and colloidal instability, these pose limitation in terms of their use in diagnosis and therapy for both cancer and other diseases. Moreover, QDs still have questions pertaining to their cytotoxicity and their size increase after coating. The materials also show impressive results in imaging tumor neovasculature which is done late after clinical diagnosis of the development of the cancer. Gold nanorods have very high absorption spectra in the NIR; this is where light penetration into the tissue is very high, around 10 cm deep. Gold nanorods relative to quantum dots are easy to synthesize, they have tunable optical properties, and they can also be multifunctional [11].

### 2.2. Porphyrins

Porphyrins and metalloporphyrins are tetraazamacrocyclic compounds found in nature, and the compounds participate in important biological processes such as photosynthesis. Importantly, the ability of a porphyrin photosensitizer to be located in a biological medium depends strongly on their peripheral substituent groups as well as their axial ligand; the two factors determine solubility, chemical affinity, redox potential, and other properties [148–150].

#### 2.2.1. Synthesis of Porphyrin and Characterization

**Figure 12:**
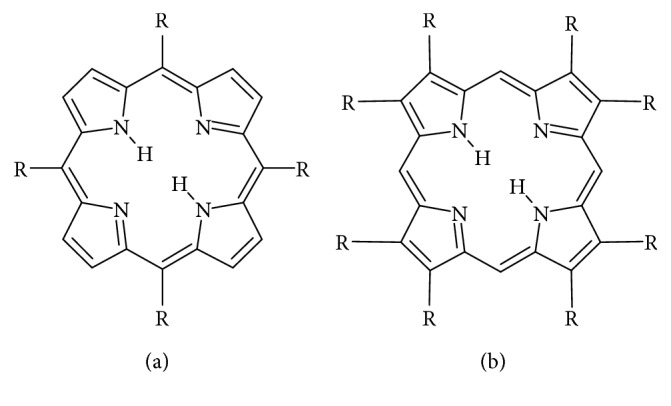
Structures of the (a) mesosubstituted and (b) *β*-substituted porphyrins.

Porphyrins exist as two types, *β*-substituted and mesosubstituted porphyrins, as illustrated in Figure 12 [151], where the *β*-substituted porphyrins mirror naturally occurring porphyrins while mesosubstituted are synthesized. Mesosubstituted porphyrins are widely used in biomedical and material chemistry [152].

The synthesis of the mesosubstituted porphyrins from nonporphyrins follows basic steps which are seen in the methods described here. The first synthesis was performed by Rothermund in 1936, and the method starts with the reaction of an aldehyde and pyrrole with both reactants at high temperature and concentration in a bomb reactor with no added oxidant. The addition of zinc acetate to the reaction only increases the yield of tetraphenylporphyrins two-fold, but this is not observed with any other porphyrins [153–157].

**Figure 13:**
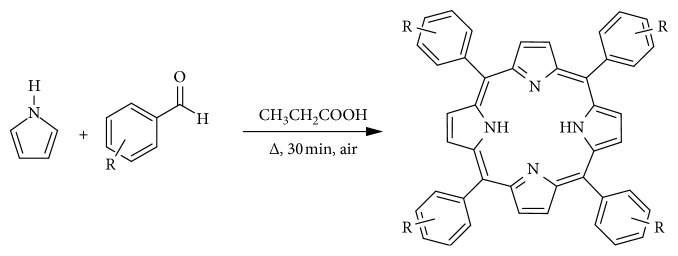
Synthesis of mesosubstituted tetraphenylporphyrin in open air [158].

**Figure 14:**
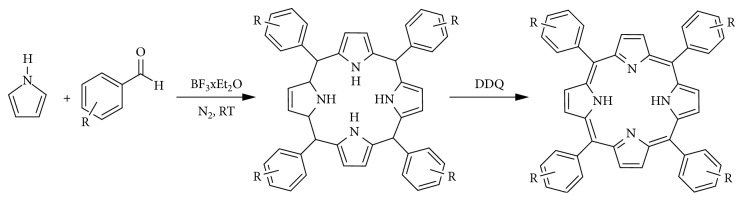
Schematic method as by Linsdey in 1987 [159].

Another method was developed by Adler and Longo so as to modify the Rothermund method by increasing the yields. The method was developed around the 60s and uses high temperature and concentration, but unlike the Rothermund method, it takes place in the presence of air under reflux in propionic acid. The method allows for synthesis of various porphyrins at relatively higher yields but polymerized pyrrole yield also increases, hence reduced porphyrin yields because it contaminates the product. The porphyrin can be isolated in two ways: treating with DDQ (2,3-dichloro-5,6-dicyano-1-4-benzoquinone) and refluxing with toluene or by using column chromatography. Scheme 1 illustrates the synthesis by the Adler–Longo method [158]. Figures 13 and 14 show a reported method scheme as performed by Linsdey in 1987.

**Figure 15:**
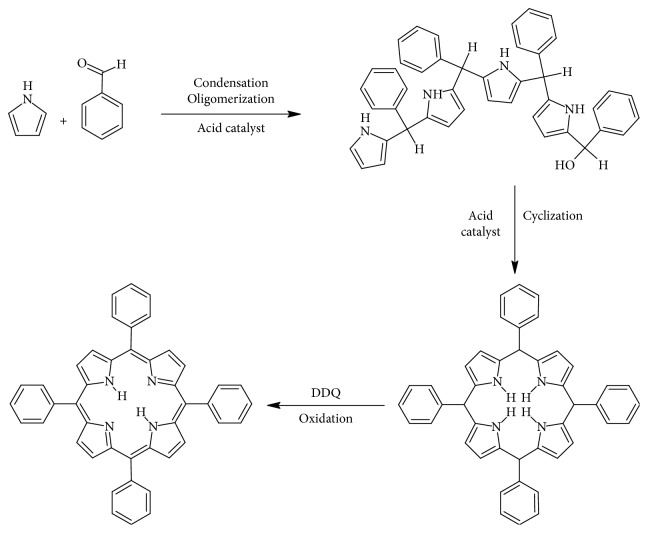
Reaction transformation from an aldehyde to a porphyrin.

The Adler–Longo reaction is used to react substituted benzaldehydes with pyrroles to produce the corresponding porphyrins in yields of about 20%. The method provides crystalline and pure product relative to the Rothermund reaction product, and it can also be used with different aldehydes to produce porphyrins but it poses certain problems; the high temperatures and concentrations hinder the synthesis of porphyrins with sensitive functional groups [144, 160, 161]. The reaction method as established by Alder, Longo, and Shergalis shows that before the porphyrin is cyclised, it goes through a carbinol step as shown in Figure 15 [162].

**Figure 16:**
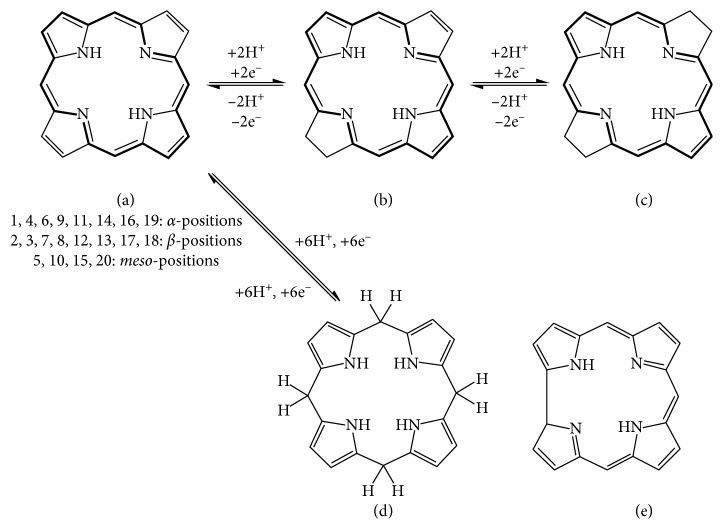
(d) The tetrapyrole derivative porphynogen, a synthetic precursor of the porphyrin ligand. (a) Porphyrin. (b) Chlorin. (c) Bacteriochlorin. (d) Porphyrinogen. (e) Corrin.

Due to the drawbacks brought by the Adler–Longo method, the Linsdey method was developed. The method synthesizes porphyrins under milder conditions and is a two-step one-flask reaction. The method enhances the Adler–Longo method, in that porphyrins that cannot be synthesized by the latter can be synthesized by the Lindsey method; this method can yield porphyrins from some sensitive aldehydes. In this method, pyrrole, benzaldehyde, triethylorthoacetate, and a water quencher are stirred at room temperature at equimolar concentrations with boron triflate in dichloromethane (DCM). After an interval of 30–40 minutes, formation of porphyrinogen occurs. DDQ is added to oxidise the porphinogen to produce the porphyrin in a yield of 30–40%.  Figure 16 [163] shows the reaction path from an aldehyde to a porphyrin.  Figure 14 illustrates a reaction scheme for the formation of porphinogen [164–171].

#### 2.2.2. Modifications in the Structure

*(1) First-, Second-, and Third-Generation Photosensitizers*. Photofrin has intrigued synthesis of a lot of other photosensitizers such as photoheme which is used to treat lung, skin, and breast cancer, and these are the first-generation photosensitizers [172–184]. Second-generation photosensitizers were developed so as to improve on first-generation photosensitizers. These photosensitizers are chemically pure and absorb light at around 650 nm, and they have relatively less skin photosensitivity. A lot of research has been conducted and shows that the sensitising efficiency of a compound increases with decreasing polarity [185]. The use of Photofrin has triggered the synthesis of more porphyrin-based photosensitizers mainly to produce the ideal photosensitizer. Numerous new porphyrin-based photosensitizers have been produced: first- and second-generation photosensitizers including hematoporphyrin monomethyl ether (HMME), photocarcinogen (PsD-007), second-generation hematoporphyrin derivatives (HiPorphyrin), and 5-ALA (aminolevulinic acid hydrochloride) [186]. The activation of a porphyrin and its derivatives by light triggers its relaxation to the ground state in three ways: nonradiative decay, emitting a photon, or by the transfer of energy [187–189]. Photofrin, which is the purified form of hematoporphyrin, was the first accepted photosensitizer for PDT, for the treatment of various cancers. Photofrin has certain properties required for an ideal photosensitizer but it has a few challenges; it has a weak long wavelength at 630 nm which is below the wavelength for maximum tissue penetration of deep tumors; secondly, it has a long photosensitivity to the skin. The surface of porphyrins can also be modified by different methods including surfactant-resistant, ionic, mixed porphyrin, sonic cation-assisted, and metal coordination self-assembly [190–192].

#### 2.2.3. Modification of Porphyrins

Porphyrins can be modified by expanding their pi-electron conjunction. This is achieved by increasing the heterocyclic rings or bridging carbons around the porphyrin framework. The resulting chromophores absorb strongly in the red region, 650–800 nm as opposed to the 18 pi-electrons in normal porphyrins [193–195]. Modification to the porphyrin structure enhances the absorbing ability of the porphyrin; for example, the reduction of the ring produces a ring of chlorine which absorbs around 660 nm in the red spectra. This allows for the modulation of the light-activated analysis [196].

#### 2.2.4. Porphyrin in Photosensitization

The use of two-photon imaging over conventional imaging has many advantages including the deeper light penetration, 3D image capability, low background fluorescence, and reduced damage to the surrounding tissue. The use of gold nanorods as contrast agents of two-photon imaging is very essential due to the fact that they exhibit high two-photon luminescence which is 58x more than that of a rhodamic molecule [197, 198]. In the two-photon imaging, mesoporous silica nanoparticles have been used, so as to evade the shortcomings of photosensitizer delivery systems; they are used as carriers in chemical catalysis, drug delivery, and cell labelling. Mesoporous silica nanoparticles have high pore volume and surface area, hence ease for the production of singlet oxygen which is easily released from the matrix. The structures also show high biocompatibility and tolerance to most organic solvents; these compounds are also easily functionalized for targeting tumor cells *in vivo* [199–206].

#### 2.2.5. Application of Porphyrins

The application of porphyrins is based on their peculiar characteristics including that they have rigid and planar geometries, photothermal, and spectroscopic properties that can be tailored readily; they are multifunctional, biocompatible, and serve as electron donors [207, 208].

**Figure 17:**
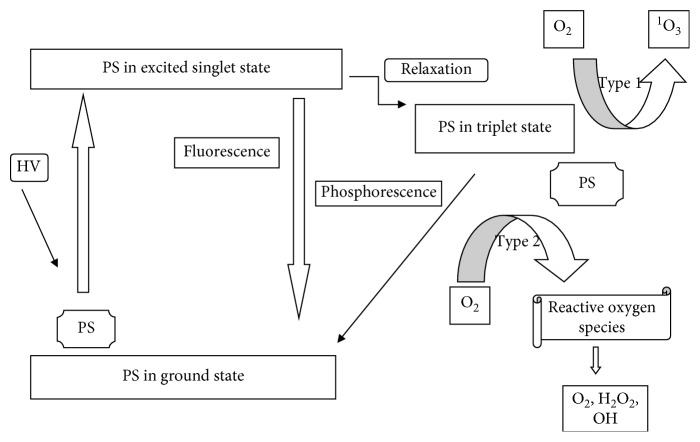
Schematic representation of a photosensitizer before and after excitation.

Photodynamic therapy is used for the treatment of tumor and malignant diseases. Its basis is on the administration of the photosensitizer which is followed by irradiation of light at a specific wavelength, and this results in reactive species like radicals or singlet oxygen which ultimately destroy the tumor cells; this phenomenon is illustrated in Figure 17 [209]. The selectivity of PDT depends on the concentration of the photosensitizer, on the normal and tumor cells, and on the exposure to light of the side being treated [210].

New antibacterial approaches include the use of photosensitizers activated with visible light; as in PDT, the photosensitizers accumulate in microbial cells to induce phototoxic reactions. The excited PS in the triplet state in the presence of oxygen induces the production of reactive oxygen species (ROS), which then continually induces further reactions in the bacterial cell wall and the lipid membranes. Maich [211] reports that a specific photosensitizer with the ability to only target the bacteria without causing harm to the surrounding environment is still a challenge [212].

The low-dark toxicities of porphyrin macrocycles and the fact that porphyrins selectively localize on a wide range of tumors have led to them being one of the best photosensitizers relative to other macrocycles. Porphyrins and compounds based on porphyrins strongly absorb in the visible part of the optical spectrum; they are noncytotoxic in the dark, have high chemical stability, have high affinity for serum proteins, can be modified to have favorable pharmacokinetic properties, and are stable when complexed with different metals but still maintain their *in vivo* tumor localization properties [213, 214].

Most photosensitizers used in PDT and fluorescence diagnosis (FD) are based on the porphyrin structure because generally porphyrin accumulates on cancer cells as opposed to the surrounding nearby healthy cells and because their fluorescensing properties are used for the detection of the cancer cells. Therapy resulting from the photosensitizers is based on the production of singlet oxygen when the photosensitizer is exposed to light. A major setback however for PDT is that it does not work best on distal metastasis [215].

Porphyrins and their analogs are adopted in many nanotechnologies due to their supramolecular design, flexibility, robustness, and unique photophysical and chemical properties and are used in technologies such as catalysts, sensors, molecular electronics and solar energy upconversion [216]. Porphyrins also have a visible light absorption and synthetic versatility, thus can be applied to the optoelectronics and act as photosensitizers [217]. Engelmanm et al. [218] demonstrates the use of two cationic porphyrins to understand factors influencing the binding of the porphyrins to liposomes and mitochondria and how efficient their photodynamic reaction is in enthrocytes. The results showed that binding and photodynamic efficiency were inversely proportional to the number of positively charged groups but directly proportional to *n*-octanol/water partition coefficients [219, 220].

#### 2.2.6. Encapsulation of Gold Nanorods with Porphyrins

The use of nanoparticles has been very impressive, but when used by themselves, they pose certain shortcomings; for example, when entering the blood system, macrophages and phagocytes readily passivate them leading to accumulation in the liver, spleen, and lymphatic system; this leads to toxicity, causes oxidative stress, and draws out an immune response [220]. Theranostic agents to be developed have to be the ones that contain both therapeutic and imaging properties. A lot of chemotherapeutic agents cannot be used as theranostic agents because they comprise less drug than the imaging doses; moreover, they are not tumor specific; antibodies on the other hand have the ability to be used as theranostic agents but they are relatively expensive. Porphyrins and derivatives however have the ability to fluorescence when excited by light, hence allowing for imaging before or after therapy; these molecules also allow for the attachment of tumor-targeting moieties at peripheral positions for the development of tumor-specific agents [221].

**Figure 18:**
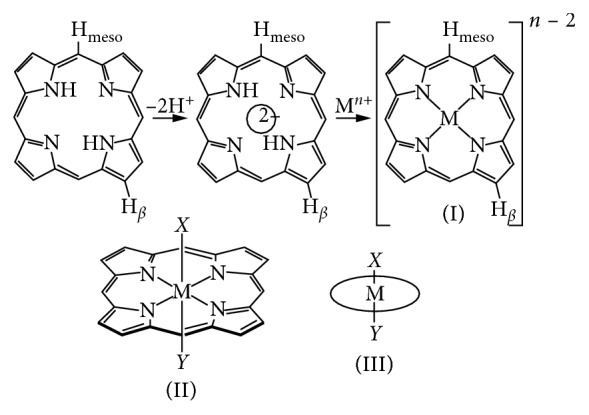
Schematic mechanism of a porphyrin complex presenting a tetra-coordinated metal in its tetrapyrolic core. (i) Metal *trans*-coordinated to two ligands. (ii) Simplified presentation.

Porphyrins have a very rigid highly stable macrocyclic structure; hence, they are used as ligands in chemistry and biology; moreover when coordinated to metals, they give new characteristics. Metal-porphyrin chemistry is very important in biomimetic and chemical applications [222, 223]. The removal of the internal protons from the “free base” porphyrin gives a tetradentate chelating dianion molecule with an ability to coordinate a metal at the central cavity, as demonstrated in Figure 18 Research reports show that porphyrins can coordinate with lanthanides, some actinates, and a few main group metals [224].

Metals bounded to porphyrins can be in a range of oxidation states; −2, −6, (*d*^0^ − *d*^10^) spin; *S* = 0 to *S* = 5/2; and a coordination ranging from 4 to 8. Restriction on the metal coordinated with the porphyrin confines them to only have two mutually *trans*-coordination sites as shown in Figure 18 [225]. Peripheral substitution or enhancement of porphyrin is an approach for modifying the steric and electronic factors of metals but rearranging the meridonal or facial types is not possible.

The versatility of the porphyrin ligand has led to a very large number of designed porphyrin complexes. Paths followed for the insertion or *M* − *L*_n_ fragment have been explored and depend on the nature of the metal source. Reactions of porphyrins and metals can be of different paths which basically depend on the previous oxidation state of the metal and coordination, reduction, or oxidation of the metal.

The noncovalent interaction of porphyrins and their derivatives are very important in biological systems. Derivatives that possess a positive charge on the mesoposition interact well with DNA, nucleotides, and a lot of aromatic substrates [226]. Neutral porphyrins are reactive but the dications are inert. Metalloporphyrin reactions depend on a number of factors which influence the nature of the reaction, the rate at which the metal incorporates is decreased by the electron withdrawing group in the *β*-substituent and the solvent in use also influences the reactions [227].

Two photon-imaging systems have attracted a lot of research due to a number of advantages including the fact that there is enhanced penetration of light in the tissue or cells. The systems also have low fluorescence, and the photodamage on the living cells is reduced with their use. Using photodynamic therapy to treat cutaneous maligneous and intraperitoneal tumors has been researched to be one of the excellent pathways to use. The method however uses photosensitizers which currently have a lot of pitfalls, problems that can be solved by development of a new improved photosensitizer or enhancing the already existing ones. The aim will be to increase the cytotoxicity, selectivity, and protection against degradation.

**Figure 19:**
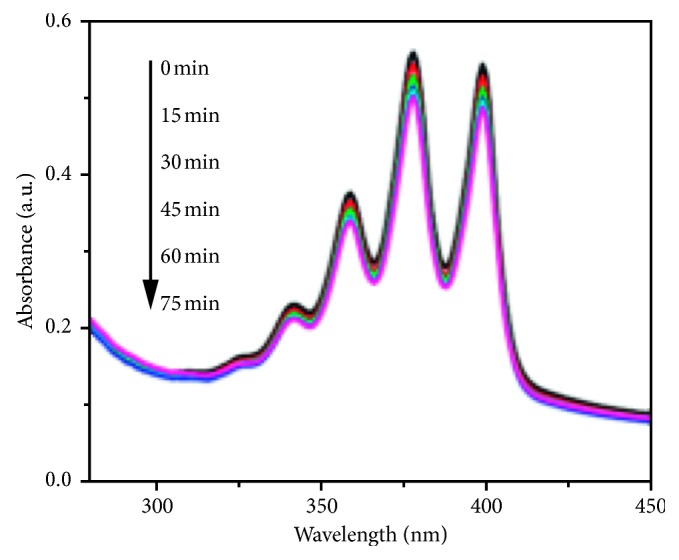
Graph showing a decrease in absorption of a composite AuNRs/mSiO_2_HP with ADBA with increased illumination time.

The encapsulation of porphyrin with silica or an additional entity protects it from biological degradation. Multifunctional nanoparticles have been shown to produce more singlet oxygen than the porphyrin on its own. An example is illustrated by Zhao et al. [228] by using a two-photon imaging system with high photosensitisation and varying different image contrast agents, photosensitizers, and carriers. Here, a multifunctional nanocomposite (AuNRs/mSiO_2_HP) was designed and used. The nanoparticles comprise a gold nanorod core with a porphyrin-doped mesoporous silica shell. Results included the fact that the triplet oxygen production was enhanced with the addition of the silica coating as opposed to pure porphyrins. These were monitored by the incorporation of ADBA (anthracenediyl-bis(methylene)dimalonic acid, which readily reacts with newly produced singlet oxygen to produce endoperoxide. Endoperoxide on the other hand decreases the ADBA absorption which is around 350 nm. The absorption spectra of the composite material embedded with ADBA can be seen in Figure 19 [228].

Research has shown that enhancing the surface of gold nanorods enables them to be versatile, hence can tune their properties to desired use. Using alkyl thiol to encapsulate the gold not only anchors the nanorod but also enhances the stability of the nanorod and fills the space potentially between the gold nanorod and bulky porphyrin molecule so as to provide space to insert C_60_ fullness molecules which increase stability to the porphyrin-gold nanorod hybrid structure. Work done by Xue and colleagues [229] showed that protection of nanorods with a monolayer of thiols and porphyrins has very interesting result.

**Figure 20:**
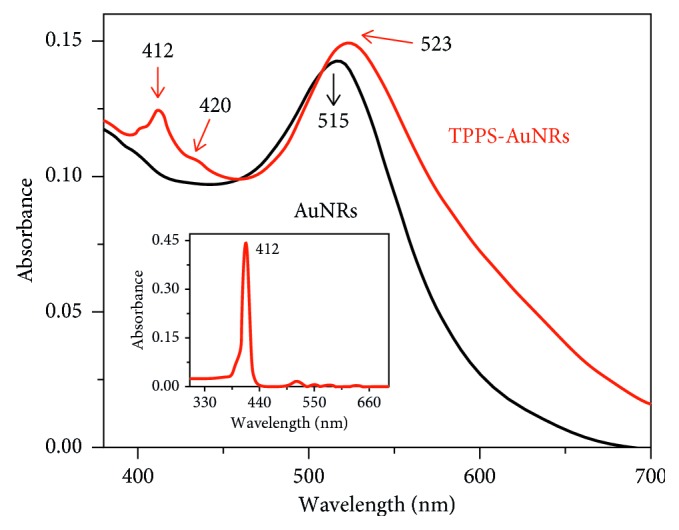
UV-Vis spectrum of TPPS-AuNRs and AuNRs.

Modification of gold nanorods can also be done by incorporation of a porphyrin with an antitumor drug, for example, doxorubicin (DOX) used usually for the treatment of a number of cancers. The goal is to increase and improve on the multidrug resistance, nanotargeted delivery, and the toxicity of the drugs. Multifunctional nanocomposites of meso-tetrakis-(4-sulfonatophenyl) porphyrin (TPPS), gold nanorods, and DOX (DOX@TPPS-AuNRs) were synthesized by Bera and his colleagues [230]. The resulting composite material showed improved cellular uptake by the cells and showed no cell toxicity.  Figure 20 [231] shows UV-Vis spectrum of the composite which significantly shows the SPR (surface plasmon resonance) of TPPS-AuNRs at 523 nm, and one of the pure AuNRs is at 525 nm in aqueous solution. The noticeable peak broadening was observed due to the presence of porphyrin which meant that there was a strong association of the gold surface to the porphyrin.

The excellent imaging and sensing properties shown by AuNRs qualify them as excellent for photothermal therapy; on the other hand, porphyrin and derivatives show excellent properties as photosensitizers due to the fact that they have more than one absorption band in the near-infrared region; hence, they can be manipulated to be used to penetrate deeper into the tissue for photodynamic therapy. Photodynamic therapy shows minimal invasion to tissues while photothermal therapy is specific to tumor cells or affected area. Photodynamic therapy limitations are still major issues up to date especially based on singlet oxygen production, selectivity to the target tissue, and the concentration so as not to damage the normal cells [231].

The agglomeration of AuNRs with porphyrins is to increase the specificity and targeting. The system incorporates two modalities: the photothermal and photodynamic stability which will hence increase the chance of tumor and bacterial diseases to be treated. Incorporation of PTT and PDT as a dual technology for cancer and bacterial infections is likely to ensure selectivity and improved efficacy to the system. This study researches and further experiments on both areas with a view to finding a more specific and less invasive combination technology.

### 2.3. Phototherapy

#### 2.3.1. Applications of Photodynamic Therapy

Photodynamic therapy (PDT) is the use of a photosensitive agent on tissues to treat cancer or bacterial infections followed by photoirradiation. This is a clinical treatment used in different diseases [12, 232–234]. It takes place by the administration of a photosensitizer in the body of a patient to accumulate on the tumor. The tumor is irradiated with biothermal light (500–650 nm), leading to an excited photosensitizer [149, 235]. Combination of the excited triplet photosensitizer and molecular oxygen results in a singlet oxygen [^1^O_2_] which is the main mediator of the destruction of the cell induced by PDT. The generation of the [^1^O_2_] has a very short life span and a limited diffusion rate, resulting in photo-oxidation of the tumor [236, 237]. A lot of photosensitizing agents have been formed but they have not been tested clinically due to factors such as (a) poor selectivity in terms of the target tissue and the healthy tissue, (b) the absorption spectra at short wavelengths, and (c) high accumulation rates in skin [236].

The use of photodynamic therapy extends to research of quantum dots, porphyrin, and micelles as photosensitizers. The discovery of PDT in the 1900s erupted as a cancer, HIV coronary heart, and psoriasis treatment agent [237–239]. PDT is a modal system which provides binary selectivity, which is accomplished by the increase in accumulation in the target tissue and limiting irradiation. The photosensitizer used in PDT has to be cytotoxic to damage only the affected tissue. An enhancement can be made by attachment to the part which has high affinity to the target tissue [240–243].

The tendency of a photosensitizer used in PDT to accumulate only in cancerous cells or tissue and not on the normal tissue is apparently explained by two reasons. It is taken up mostly by hyperproliferative cells than by normal resting cells, and their uptake by neovascular endothelial cells are accelerated, both of which characteristics can be seen in solid tumors [244, 245].

Photodynamic therapy is used to deactivate microorganisms by not leading to the selection of mutant-resistant chains compared to traditional antibiotics [246, 247]. The use of PDT has been improved by the incorporation of nanoparticles which have been used as delivery agents of the photosensitizer or to improve the inactivation kinetics.

Inorganic nanoparticles such as TiO_2_ can also be used and have the capability of inactivating microorganisms [248]. Various developments of tumor target photodynamic therapy have been made; but until now, there is no universal approach due to spontaneous properties of tumors. For the ideal PDT development, the PDT has to be tailored, have an appropriate target strategy, and carefully select the tumor type and the stage of the disease [249].

Photodynamic therapy fundamentally relies on the accumulation of a photosensitizer of a tumor cell or tissue after administration [250, 251]. This phenomenon involves the production of reactive oxygen and free radicals which are cytotoxic, and the main source of the photobiological activity is however the [^1^O_2_] which causes damage of cells either by apoptosis or recrosis [252, 253].

The use of supraparamagnetic iron oxide nanoparticles as MRI contrast agents for brain tissue has been proven very effective. Nanomaterials are used as delivery agents across the blood-brain barrier and to specifically transport drugs to cellular compartments such as the nucleus [254, 255].

Antimicrobial photodynamic therapy (aPDT) has been an effective treatment to damage bacteria. Enhancing antimicrobial photodynamic therapy (aPDT) with nanoparticles is a growing field to minimize the use of antibiotics to treat infectious diseases. The technology has many advantages including the increment of [^1^O_2_] yield of the photosensitizer [256]. A model was made by Hashimoto et al. [257] based on a burned wound and blood stream infection for the verification of aPDT. Two wavelengths were tested *in vitro*, blue and LEDs on a pathogen with resistance to antibiotics and using HB: La^3+^ as a photosensitizer. Experiments were also done *in vivo* on mice; both experiments proved that aPDT can be used in the treatment of bacteria in burned patients using PDT [258].

#### 2.3.2. Mechanism of PDT Cytotoxicity

Singlet oxygen has a relatively short lifetime (see Figure 15), i.e., 810–320 nanoseconds, thereby making its diffusion to be limited to 10–55 nm in cells, the implication being that cells only near the photosensitizer will be damaged [259, 260].

#### 2.3.3. Photodynamic Therapy Using Porphyrin

Photodynamic therapy is the recommended method to treat cancer in the developing countries due to its relative inexpensiveness and user-friendliness [260]. In addition, Chen and Zhang [261] proposed an innovation in which luminescent nanoparticles *in vivo* were used instead of supporting the PDT with external light. The use of these nanoparticles apparently reduces potential damage to the surrounding healthy cells with reasonable costs. The method normally combines both radiotherapy and photodynamic therapy through the attachment of luminescent nanoparticles with photosensitizers such as porphyrin [262]. No external light source is required for this treatment but rather exposure of the photosensitizer to ionizing radiation. The combination enhances the efficiency to damage cancer cells and reduce radiation. Nonetheless, the radiation has to be sufficient enough to produce enough light for PDT, and the prediction of level radiation is usually difficult [263, 264].

#### 2.3.4. Applications of Photothermal Therapy

Photothermal therapy (PTT) is a treatment minimally invasive which occurs by conversion of photon energy to heat energy [265]. The selectivity of the therapy is obtained by the control of incident radiation used and accompanied by the conduct of some proactive molecules or nanoparticles. The photoexcitation relaxation of the particles induces heat transfer to the surrounding affected environment [266, 267].

Nanomaterials with photothermal effects are of great interest to researchers especially in the biological imaging and therapeutics. Nanorods and nanocages possess photothermal properties, hence their use in MRI imaging, infrared thermal imaging, and photothermal ablation of cancer tissues [19, 268–274].

#### 2.3.5. Photothermal Therapy Using Gold Nanorods

**Figure 21:**
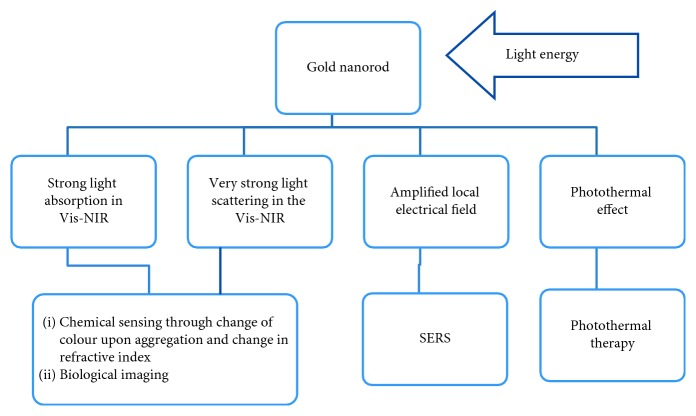
AuNRs effects and applications [290].

Gold nanorods (AuNRs) attracted researchers due to their easy and quick synthesis and simplified bioconjugation, high and strong absorption through their cross-section, and the fact that their optical extinction is tuneable [275–279]. Varying the aspect ratios of the AuNRs influences longitudinal plasmon absorbance shift throughout the visible region [95, 280–285]. AuNRs scatter light through paths of extensive elastic scattering and intensive light scattering of molecular vibrations near the metal surface. The effect is called surface-enhanced Raman scattering (SERS) which is a result of oscillations around the nanoparticle upon radiation [286–289]. These phenomena qualified gold nanorods as chemical sensors as indicated in Figure 21.

In addition, many researchers used gold nanorods as photothermal agents to damage *Pseudomonas aeruginosa*, a Gram-negative pathogen [291–295]. Their work represented gold nanorods which absorbed light at 785 nm and were conjugated to antibodies specific to bacteria. The results positively indicated 75% destruction of infected cells.

#### 2.3.6. Photothermal and Photodynamic Therapy

Recent research has been on nanocomposites based on plasmonic nanoparticles and fluorescent or photodynamic dyes. These are then used for simultaneous therapy and diagnosis [95, 282, 283]. Metal nanoparticles used in the methods are combined with photodynamic drugs using cross-linking procedures or by the use of electrostatic interactions [296–300].

**Figure 22:**
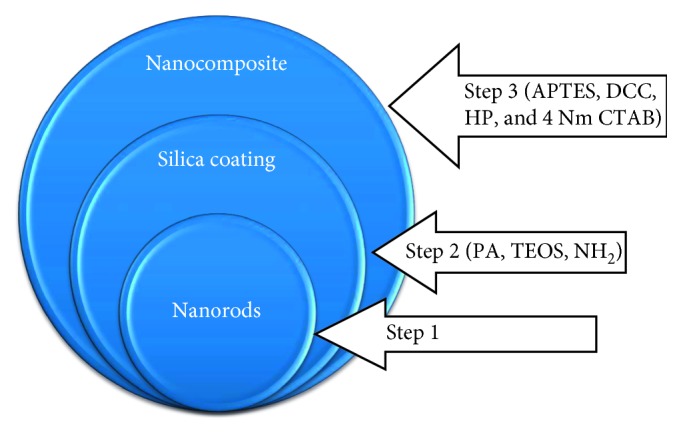
Scheme of the fabrication of AuNRs/SiO_2_-HP. IPA, isopropyl alcohol; TEOS, tetraethyl orthosilicate; APTES, 3-aminopropyltriethoxysilane; HP, hematoporphyrin; DCC, dicyclocarbodiimide; CTAB, cetyltrimethylammonium bromide [302].

The combination for simultaneous therapeutic purposes was fairly researched. Teretyvk et al. [301] incorporated PDT and PTT by the use of fabricated AuNRs/SiO_2_-HP composite nanoparticles which successfully decreased large tumor volumes and to damaged solid tumor cells, using the steps (see Figure 22) to fabricate the gold nanorod. On the other hand, Liu et al. [303] synthesized MoS_2_ nanosheets which are water-soluble and functionalized them with lipoic acid terminated polyethylene glycol (LA-PEG) to obtain MoS_2_-PEG. These are apparently stable in physiological solutions and also have the ability to be loaded with photosensitizers. Results of the nanocomposites formed using Ce_6_ as a photosensitizer in PTT and PDT *in vivo* showed an enhanced tumor necrosis. Jang et al. [304, 305] proposed that for PTT and PDT to work effectively, the distance between the gold nanorod and the photosensitizer must be manipulated. They used an AuNRs-AlPcS_4_ composite, and his results proved that the nanocomposite was effective for NIR fluorescence imaging for affected cancer sites and to improve *in vivo* therapeutic efficacy [298, 306–310].

## 3. Conclusions and Future Developments

The excellent imaging and sensing properties shown by gold nanorods qualify them as excellent for photothermal therapy. On the other hand, porphyrin and derivatives show excellent properties as photosensitizers due to the fact that they have more than one absorption band on the near-infrared region; the phenomena allow for the penetration deeper into the tissue, and hence, they have excellent use in photodynamic therapy. Photodynamic therapy shows minimal invasion to tissues while photothermal therapy is specific to tumor cells or affected area.

Photodynamic therapy limitation is still an issue up to date especially based on singlet oxygen production, selectivity to the target tissue, and the concentration so as not to damage the normal cells. Despite efforts of several researchers, an ideal photosensitizer is still not discovered, one that is safe and selective and the other that does not cause skin photosensitivity and pain. Many photosensitizers used recently have very low water solubility and tend to aggregate under physiological conditions; moreover for clinical applications, the target tissue is still not recognised.

Incorporation of PTT and PDT as a dual technology for cancer and bacterial infections ensures selectivity and improved efficacy to the system. Previous attempts have been made using and varying different porphyrin or derivatives for photodynamic therapy or using different noble metals or carbon nanotubes for photothermal therapy or incorporating the two therapies but no ideal photosensitizer or an ideal specific drug which damages only the cancer cells has been discovered. This review is aimed at giving and overviewing of the studies made on the application of photothermal and photodynamic therapy by encapsulating gold nanorods with porphyrin for better cancer and bacterial therapy. A thorough research and further experimentation will be made on both so as to find a more specific and less invasive technology.

## Figures and Tables

**Table 1:** Summary of properties, synthesis, characterization, and applications of AuNRs

Synthesis	Characterization	Limitation	Application
Wet chemical synthesis [100–102], template method [103–105]	SEM, TEM	Limited yield due to the CTAB bilayer	Sensing, photothermal therapy, imaging
Electrochemical route [106–108]	TEM	N/A	Sensing, photothermal therapy, imaging
Seed-mediated method [109–111]	TEM, ETEM	N/A	Sensing, photothermal therapy, imaging
